# Structural Studies and Investigation on the Activity of Imidazole-Derived Thiosemicarbazones and Hydrazones against Crop-Related Fungi

**DOI:** 10.3390/molecules181012645

**Published:** 2013-10-14

**Authors:** Débora C. Reis, Angel A. Recio Despaigne, Jeferson G. Da Silva, Nayane F. Silva, Camila F. Vilela, Isolda C. Mendes, Jacqueline A. Takahashi, Heloisa Beraldo

**Affiliations:** Departamento de Química, Universidade Federal de Minas Gerais, Belo Horizonte 31270-901, MG, Brazil

**Keywords:** imidazole, hydrazones, thiosemicarbazones, antifungal activity

## Abstract

New imidazole derived thiosemicarbazones and hydrazones were prepared by condensation of 4(5)-imidazole carboxaldehyde, 4-(1*H*-imidazole-1-yl)benzaldehyde and 4-(1*H*-imidazole-1-yl)acetophenone with a thiosemicarbazide or hydrazide. All compounds were characterized by quantitative elemental analysis, IR and NMR techniques. Eight structures were determined by single crystal X-ray diffraction. The antifungal activities of the compounds were evaluated. None of the compounds exhibited significant activity against *Aspergillus flavus* and *Candida albicans*, while 4(5)-imidazolecarboxaldehyde thiosemicarbazone (ImT) and 4-(1*H*-imidazole-1-yl)benzaldehyde thiosemicabazone (4ImBzT) were highly and selectively active against *Cladosporium cladosporioides*. 4(5)-Imidazolecarboxaldehyde benzoyl hydrazone (4(5)ImPh), 4(5)-imidazolecarboxaldehyde-*para*-chlorobenzoyl hydrazone (4(5)Im*p*ClPh), 4(5)-imidazolecarboxaldehyde-*para*-nitrobenzoyl hydrazone (4(5)Im*p*NO_2_Ph), 4-(imidazole-1-yl)acetophenone-*para*-chloro-benzoyl hydrazone (4ImAc*p*ClPh) and 4-(imidazole-1-yl)acetophenone-*para*-nitro-benzoylhydrazone (4ImAc*p*NO_2_Ph) were highly active against *Candida glabrata*. 4(5)Im*p*ClPh and 4(5)Im*p*NO_2_Ph were very effective against *C. cladosporioides*. In many cases, activity was superior to that of the reference compound nystatin.

## 1. Introduction

The imidazole nucleus is well known to play an important role in living organisms since it is incorporated into the histidine molecule and many other important biological systems. Imidazole derivatives are the most used class of antifungal drugs [1], being active against pathogenic and nonpathogenic fungi [2]. Due to their antifungal properties imidazole-derived compounds have been used in agriculture as effective ingredients for controlling plants pests. Imidazole derivatives are employed in the control of spoilage microorganisms or organisms potentially harmful to man, in the protection of wood against fungi and also in food storage [3]. In addition, imidazole compounds were reported to be active against *Fungi imperfecti*, *Basidiomycetes*, *Ascomycetes* and *Oomycetes*. Outstanding activity was observed against powdery mildews (e.g., *Erysiphenecator*) and leaf spots (e.g., *Mycosphaerella spp.*). Furthermore, these compounds are effective against phytopathogenic *gram*-negative (e.g., *Xanthomona spp.*, *Pseudomonas spp.*, *Erwinia amylovora*, *Ralstonia spp.*) and *gram*-positive bacteria and viruses (e.g., tobacco mosaic virus) [4].

*A. flavus* is a worrying plant pathogen since it can produce aflatoxins on certain crops like oilseeds, corn and nuts (e.g., peanuts). Fungi unknown to produce aflatoxins can also cause agricultural damages, like *C. cladosporioides* that acts in the post-harvesting spoilage of fruits and vegetables. In vineyards, *C. cladosporioides* can also cause grape rot [5]. Both species have been reported to be among the major species normally found in several commodities like soybeans [6]. These species can become still more harmful since they can be spread by air from industrial food processing environments, such as grain mills or organic waste recycling facilities [7]. In contact with humans, they act as allergenic factors, causing a series of respiratory related diseases [8]. It has been reported that *C. cladosporioides* can play a role as opportunistic fungi causing ocular infections [9]. *A. flavus* has also been reported to be an opportunistic fungus especially in immuno-compromised patients [10].

Thiosemicarbazones [11,12] and hydrazones [13] are reported as compounds which present significant antifungal activity. Their metal complexes also exhibit antifungal properties [14,15,16]. Hydrazones have previously been reported as active against some species of *Candida* sp [16,17,18]. 

In the present work we synthesized a series of imidazole-derived thiosemicarbazones and hydrazones and tested the potential of these compounds as antifungal agents to fight *A. flavus* and *C. cladosporioides*. Since pathogens like *Candida sp*. have a huge importance as a clinical target of new fungicides, we extended the screening in order to evaluate the potential of the studied compounds against *Candida albicans* and *Candida glabrata*.

## 2. Results and Discussion

### 2.1. Characterization of the Thiosemicarbazone Derivatives

**Figure 1:**
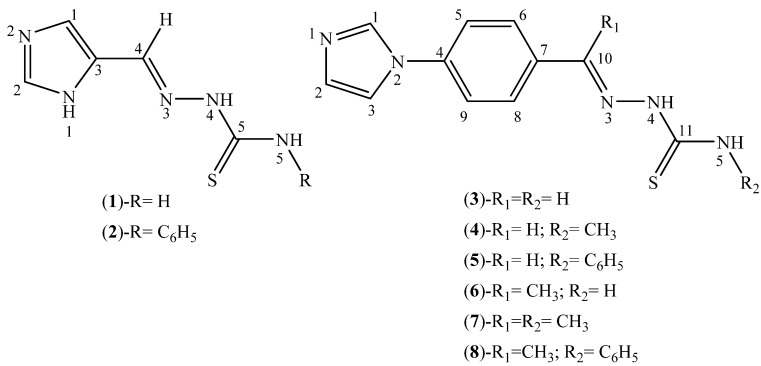
Generic representation for imidazole-derived thiosemicarbazones.

Eight imidazole-derived thiosemicarbazones (Figure 1) were obtained. Among them seven compounds (**2**–**8**) were original. Microanalyses were compatible with the proposed formulations. The infrared spectra of the thiosemicarbazones showed a very strong band at 3,446–3,145 cm^−1^ which was attributed to the ν(N–H) vibration. The ν(C=N) and ν(C=S) vibrations appeared at 1,638–1,601 cm^−1^ and 848–830 cm^−1^, respectively [19,20].

The NMR spectra of all thiosemicarbazones were recorded in DMSO-*d_6_*. The ^1^H resonances were assigned on the basis of chemical shifts and multiplicities. The carbon type (C, CH) was determined by using distortionless enhancement by polarization transfer (DEPT 135) experiments. The assignments of the protonated carbons were made by 2D hetero-nuclear multiple quantum coherence experiments (HMQC).

In the ^1^H- and ^13^C-NMR spectra of thiosemicarbazone **1** duplicated signals were observed for all hydrogens and carbons, indicating the presence of the *Z* (60%) and *E* (40%) configurational isomers in the DMSO-*d_6_* solution, as frequently occurs with thiosemicarbazones [21]. In the spectra of **2** three signals were observed for each hydrogen and each carbon. In the ^13^C-NMR the signals at δ 175.29 and δ 175.47 were assigned to C=S of the *E* and *Z* isomers, respectively, and the signal at δ 172.05 to C–SH. The proportion of *E* and *Z* isomers is 1:1, but it was not possible to determine the percentage of the *thiol* form in solution due to overlapping signals in the S–H spectral region. The thione-thiol tautomerism had been previously observed for other thiosemicarbazones [22]*.*

In the ^1^H- and ^13^C-NMR spectra of **3**–**8** only one signal was observed for each hydrogen and carbon; the chemical shifts indicate the presence of only the E isomer in solution. Generally when N5–Hb is replaced by a methyl or a phenyl group in **4**, 5, **7** and **8** the signal of N5–Ha shifts upfield. For both formyl and acetyl compounds the signals of the imidazole and benzene rings were observed in the same region.

The N4–H signals at δ 11.93–11.53 (formyl derivatives) and δ 10.66–10.27 (acetyl derivatives) indicate hydrogen bonding with the solvent [21]*.* In the spectra of the acetyl derivatives the signals of N4–H appear at lower frequencies due to the gamma-gauche effect of the CH_3_ group in C10, which makes more difficult the interaction of DMSO-*d*_6_ with N4–H [23]*.*

**Figure 2:**
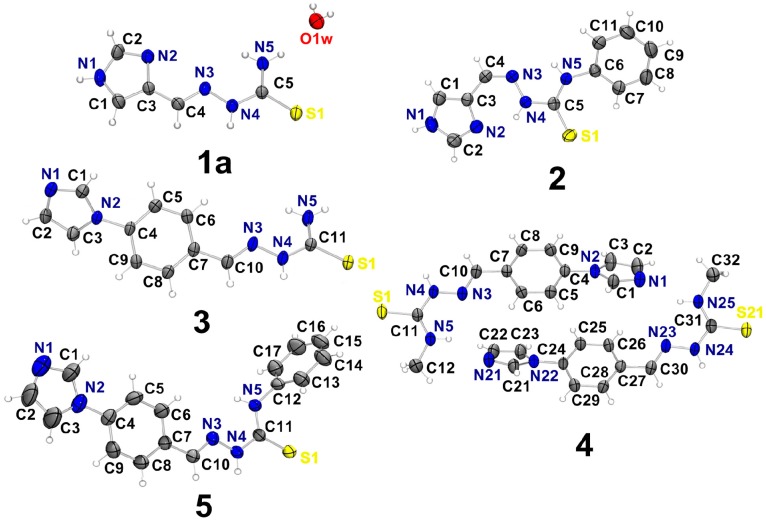
Molecular plots of ImT∙H_2_O (**1a**), ImTPh (**2**), 4ImBzT (**3**), 4ImBzTM (**4**) and 4ImBzTPh (**5**), showing the labeling scheme of the non-H atoms and their displacement ellipsoids at the 50% probability level.

Crystals suitable for X-ray diffraction studies were obtained by slow evaporation from the mother liquor in the synthesis of **1**, **2**, **3**, **4** and **5**. Interestingly, **1** crystallized with one water molecule as ImT∙H_2_O (**1a**). The crystal data and refinement results for imidazole-derived thiosemicarbazones are given in Table S1 of the Supplementary Material. The ORTEP diagrams for **1a** and **2**–**5** and the numbering scheme are shown in Figure 2. Selected bonds distances and angles are listed in Table S2. The geometric parameters for hydrogen bonds in **1a** and **2**–**5** are listed in Table 1.

Compounds **1a**, **2**, **3** and **5** crystallized in the monoclinic system with one molecule of the thiosemicarbazone per asymmetric unit, while **4** crystallized in the triclinic system with two molecules per asymmetric unit (see Figure 2). In all structures the CNNC(S)N backbone is almost planar with *rms* distance of atoms from the least-squares plane of 0.0277 Å (**1a**), 0.0137 Å (**2**), 0.0705 Å (**3**), 0.0438 and 0.0669 Å (for C10N3N4C11S1N5 and C30N23N24C31S21N25, respectively, in **4**) and 0.0311 Å (**5**). The bond lengths in the thiosemicarbazone backbone are similar to those previously described in the literature [19,20,24,25].

The thiosemicarbazone backbone of **1a** and **3**–**5** adopt the *EE* conformation in relation to the C4–N3 or C10–N3 and N4–C5 or N4–C11 bonds. The N5–H⋯N3 hydrogen bond probably hinders rotation around the N4–C5 or N4–C11 bonds and might contribute to the stability of the *E* conformation. In fact, the *EE* conformation is commonly observed in 2-formylpyridine- and 2-acetylpyridine-derived thiosemicarbazones in the solid state [26,27]. Furthermore, the *EE* conformation allows the formation of dimmers *via* intermolecular N4–H⋯S1 hydrogen bonds (see Table 1). In addition, in the case of 1a a hydration water molecule forms intermolecular hydrogen bonds (see Table 1).

**Table 1:** Hydrogen bonds geometric parameters (Å, °) for ImT∙H_2_O (**1a)**, ImTPh (**2**), 4ImBzT (**3**), 4ImBzTM (**4**) and 4ImBzTPh (**5**).

D–H…A	d(D–H)	d(H⋯A)	d(D⋯A)	< (DHA)
**1a**				
O1W–H1W⋯N2 ^i^	0.8599(10)	2.158(10)	2.950(2)	153(2)
O1W–H2W⋯N2 ^ii^	0.8599(10)	2.200(5)	3.0487(19)	169(2)
N1–H1⋯S1 ^iii^	0.86	2.58	3.3540(15)	149.6
N4–H4A⋯S1 ^iv^	0.86	2.61	3.4168(14)	157.2
N5–H5A⋯O1W ^ii^	0.86	2.46	3.2804(19)	161.0
N5–H5B⋯O1W	0.86	2.11	2.9429(19)	164.2
**2**				
N1–H1⋯S1 ^v^	0.86	2.61	3.3632(14)	147.1
N4–H4⋯N2	0.86	2.02	2.7158(18)	137.2
N5–H5⋯N3	0.86	2.12	2.5866(17)	113.9
**3**				
N4–H4 ⋯S1 ^vi^	0.86	2.60	3.4424(13)	166.4
N5–H5A⋯S1 ^vii^	0.86	2.79	3.4162(14)	130.6
N5–H5B⋯N1 ^viii^	0.86	2.12	2.9405(18)	159.4
**4**				
N24–H2⋯S1 ^ix^	0.86	2.56	3.3890(18)	162.7
N25–H25A⋯N9	0.86	2.22	2.999(2)	150.9
N4–H4⋯S21 ^x^	0.86	2.55	3.3501(19)	154.8
N5–H5A⋯N21	0.86	2.21	2.984(2)	149.2
**5**				
N4–H4⋯S1 ^xi^	0.86	2.56	3.3950(17)	164.0
N5–H5⋯N1 ^xii^	0.86	2.61	3.309(3)	139.1

Symmetry transformations used to generate equivalent atoms: i = −x + 1, y+1/2, −z + 1/2; ii = −x+1, −y + 1, −z + 1; iii = x, y − 1, z; iv = −x, −y + 1, −z; v = −x + 2, y−1/2, −z + 3/2; vi = −x + 2, −y, −z + 2; vii = x, −y + 1/2, z + 1/2; viii = x−1, −y + 1/2, z−3/2; ix = x, y, z − 1; x = x, y, z + 1; xi = −x + 1, −y + 2, −z + 1 and xii = −x + 2, −y, −z + 1.

In contrast, **2** adopts the *ZE* conformation in relation to the C4–N3 and N4–C5 bonds (see Figure 2). This probably occurs due to the presence of a weak N1-H⋯N3 hydrogen bond (see Table 1). This conformation is often observed in 2-benzoylpyridine-derived thiosemicarbazones [28].

### 2.2. Characterization of the Hydrazone Derivatives

**Figure 3:**
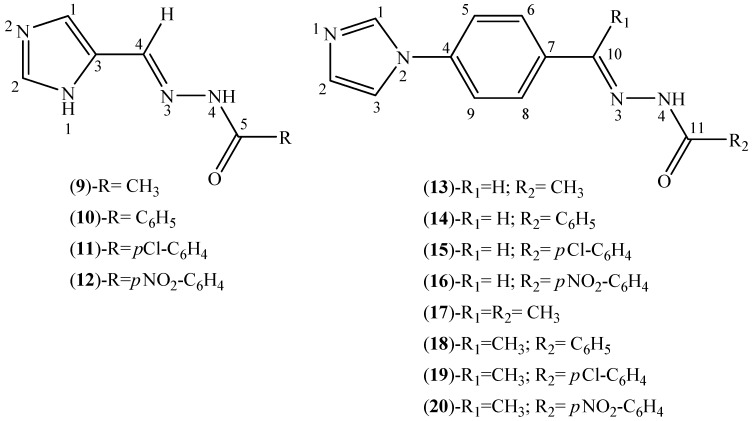
Generic representation for imidazole-derived hydrazones.

Twelve new imidazole-derived hydrazones (Figure 3) were obtained. Microanalyses were compatible with the proposed formulations. In the infrared spectra of the hydrazones the absorption at 3,240–3,139 cm^−1^ was attributed to the ν(N-H) vibration mode. Absorptions at 3,100–3,020 cm^−1^ in the spectra of compounds **9**–**12** are characteristic of N4-H⋯N2 hydrogen bonds. Very strong bands at 1,687–1,659 cm^−1^ were assigned to ν(C=O) and absorptions at 1,626–1,606 cm^−1^ were attributed to ν(C=N).

As for the thiosemicarbazones the NMR spectra of all hydrazones were recorded in DMSO-*d_6_*. Three signals for all hydrogens and carbons were found in the ^1^H- and ^13^C-NMR spectra of compound **9**, suggesting the existence of three isomers in solution. The signals of C5 at δ 171.89 and δ 171.57 were attributed to C=O of the *E* and *Z* isomers, respectively, and the signal at δ 168.35 was assigned to C–OH in the *enol* tautomer [29]*.*

The signals at δ 11.22, δ 11.03 and δ 9.76 were attributed to N1–H of the imidazole ring in the *enol*, *E* and *Z* isomers, respectively. Signals at δ 13.30 and δ 12.93 were assigned to N4–H in the *Z* and *E* isomers. The O–H signal of the *enol* tautomer is overlapped with the N4–H signal of the *E* isomeric form.

In the ^1^H-NMR spectra of compounds **10**–**12** only one signal was observed for each hydrogen. The signals attributed to N1–H were found at δ 8.38 (**10**), δ 8.39 (**11**) and δ 8.37 (**12**) and those of N4–H were found at δ 14.29 (**10**), δ 14.34 (**11**) and δ 14.54 (**12**), indicating that they are in the *Z* configuration in solution. In the ^13^C-NMR spectra of **10** and **11** one signal was verified for each carbon, confirming the existence of only one isomer in solution. The signals of C5=O were observed at δ 162.81 and δ 162.26 in the spectra of **10** and **11**, respectively. Due to the low solubility of **12** it was impossible to record its ^13^C-NMR spectrum.

The ^1^H and ^13^C-NMR spectra of compounds **13** and **17** showed duplicated signals for each hydrogen and carbon indicating the presence of two isomeric forms in solution. The signals at δ 172.00 and δ 165.67 for **13** and δ 171.82 and δ 166.13 for **17** corresponded to C11=O and C11–OH of the *keto* and *enol* tautomers, respectively [29]. The signals at δ 11.49 and δ 10.51 were attributed to N4-H in the *keto* tautomer for **13** and **17**, and those at δ 11.35 and δ 10.41 to O–H in the *enol* tautomer, respectively. The *keto* tautomers adopt the *E* configuration in solution.

In the spectra of compounds **14**–**16**, **18** and **19**, only one signal was observed for each hydrogen and carbon. The signals of N4–H appear at δ 12.22–10.84, indicating the presence of *E* configuration for these compounds in solution. The signal at δ 163.82–161.13 for **14**–**16** and the signal at δ 166.13 and δ 171.82 for **18** and **19** were assigned to C11=O. Due to the low solubility of compound **20** it was impossible to record its NMR spectrum.

**Figure 4:**
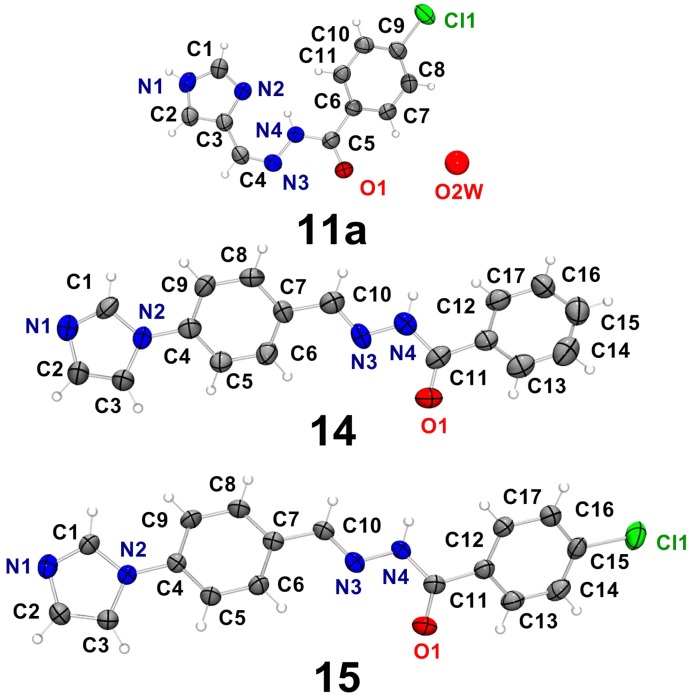
Molecular plots of 4(5)Im*p*ClPh∙H_2_O (**11a**), 4ImBzPh (**14**) and 4ImBz*p*ClPh (**15**), showing the labeling scheme of the non-H atoms and their displacement ellipsoids at the 50% probability level.

Crystals were obtained by slow evaporation from the mother liquor in the synthesis of **11**, **14** and **15**. Interestingly, **11** crystallized with one hydration water molecule as 4(5)Im*p*ClPh∙H_2_O (**11a**). The crystal data and refinement results for the imidazole-derived hydrazones are given in Table S3 of the Supplementary Material. The ORTEP diagrams for **11a**, **14** and **15** and the numbering scheme are shown in Figure 4. The selected bonds and angles are listed in Table S4. The geometric parameters for hydrogen bonds in **11a**, **14** and **15** are listed in Table 2.

The three compounds crystallized in the monoclinic system with one molecule of the hydrazone per asymmetric unit (see Figure 4). In all structures the CNNC(O)C backbone is almost planar with *rms* distance of atoms from the least-squares plane of 0.0402 Å (**11a**), 0,0476Å (**14**) and 0.0287 Å (**15**).

The hydrazone backbone of **11a** adopts the *ZZ* conformation in relation to the C4–N3 and N4–C5 bonds. The intramolecular N4–H⋯N2 hydrogen bond probably hinders rotation around C4–N3 contributing to the stability of the *Z* configuration. This conformation was also observed by us in 2-benzoylpyridine-derived hydrazones [30]. The crystal packing of **11a** is dominated by N–H⋯N and N–H⋯O hydrogen bonds (see Table 2).

In contrast, **14** and **15** adopt the *EZ* conformation in relation to the C10–N3 and N4–C11 bonds. The crystal packings of **14** and **15** are very similar and are dominated by intermolecular N4–H⋯N1 hydrogen bond (see Table 2).

**Table 2:** Hydrogen bonds geometric parameters (Å, °) of for 4(5)Im*p*ClPh∙H_2_O (**11a**), 4ImBzPh (**14**) and 4ImBz*p*ClPh (**15**).

D–H…A	d(D–H)	d(H⋯A)	d(D⋯A)	<(DHA)
**1a**				
N1–H1A⋯O1 ^i^	0.86	1.96	2.799(5)	164.4
N1–H1A⋯N3 ^i^	0.86	2.67	3.242(5)	125.4
N4–H4A⋯N2	0.86	2.00	2.698(5)	137.0
**14**				
N4–H4⋯N1 ^ii^	0.86	2.26	3.079(3)	158.8
**15**				
N4–H4⋯N1 ^ii^	0.86	2.30	3.1411(16)	166.9

Symmetry transformations used to generate equivalent atoms: i = −x + 1\2, y − 1/2, −z + 3/2 and ii = x − 1, −y + 1/2, z + 1/2.

### 2.3. Antifungal Activity

The results of the biological assays are reported in Table 3. In general, the thiosemicarbazones presented lower activity than the hydrazones. However, high activity was found for thiosemicarbazones **1** and **3** against *C. cladosporioides*, with MIC_50_ = 5.79 and 2.00 µM, respectively. These values were significantly lower than those determined for nystatin (MIC_50_ = 269 µM) which was used as positive control. Compounds **1** and **3** were selectively active against *C. cladosporioides*, since their MIC_50_ values against *A. flavus*, *C. albicans* and *C. glabrata* were much higher than those determined against *C. cladosporioides*. Derivatives **1**–**8** presented the same pattern of activity against the two *Candida* species.

From twelve hydrazones tested, only five showed some antifungal activity. Compounds **10**, **19** and **20** proved to be active against *C. glabrata*, suggesting some selectivity*.* Compound **10** was very active, with MIC_50_ < 1.20 µM. **11** and **12** were active against *C. glabrata* and *C. cladosporioides*. Both were more active than nystatin against *C*. *cladosporioides.* Compounds **12** and **20** proved to be more active than nystatin against *C. glabrata*. **12**, which bears a *p*NO_2_Ph substituent, showed higher activity against *C. glabrata* (MIC_50_ < 0.9 µM) and *C. cladosporioides* (MIC_50_ = 1.6 µM) than nystatin (MIC_50_ = 1.05 µM and > 269 µM, respectively).

Since compounds **1** and **3** have in common the presence of a primary amine group at N5 we can presume that steric hindrance at this position in the other thiosemicarbazones could lead to weaker interaction with the biological target, with decreasing of activity.

None of the tested thiosemicarbazones presented a significant activity against *A. flavus.* This is an important finding, since selectivity is a very welcome property of a given antifungal, allowing an optimum exploitation of its capacity without affecting beneficiating microorganisms present in a given crop.

Evaluation of activity for the imidazole-carboxaldehyde-derived hydrazones **9**–**12** reveals that the presence of the phenyl group in **10**–**12** significantly improves antifungal activity in relation to the presence of the methyl group in **9**. Among the imidazole-acetophenone-derived hydrazones the presence of the *p*-ClPh or *p*-NO_2_Ph groups seems to favor antifungal activity against *C. glabrata* in compounds **19** and **20**.The presence of the *p*-NO_2_Ph group in **12** and **20** and of the *p*-ClPh group in **11** and **19** seems to be very effective to increase the antifungal effect of the compounds.

**Table 3:** MIC_50_ of imidazole-derived thiosemicarbazones and hydrazones against phytopathogenic and human pathogenic fungi.

Compounds	MIC_50_ (µM)			
	*C. glabrata*	*C. albicans*	*A. flavus*	*C. cladosporioides*
ImT (**1**)	>1477.45	>1477.45	>1477.45	5.79
ImTPh (**2**)	>1019.16	>1019.16	>1019.16	1019.16
4ImBzT (**3**)	509.58	>509.58	>1019.16	2.00
4ImBzTM (**4**)	>964.02	>964.02	>964.02	>964.02
4ImBzTPh (**5**)	>777.99	>777.99	>777.99	>777.99
4ImAcT (**6**)	>964.02	>964.02	>964.02	>964.02
4ImAcTM (**7**)	>914.54	>914.54	>914.54	>914.54
4ImAcTPh(**8**)	>745.33	>745.33	>745.33	>745.33
4(5)ImMe (**9**)	>1643	>1643	1643	>1643
4(5)ImPh (**10**)	<1.20	>1167	>1167	1167
4(5)Im*p*ClPh (**11**)	15.70	1005.20	>1005.20	7.80
4(5)Im*p*NO_2_Ph (**12**)	<0.90	>964.40	482.20	1.90
4ImBzMe (**13**)	>1095.20	>1095.20	>1095.20	>1095.20
4ImBzPh (**14**)	>861.10	>861.10	>861.10	>861.10
4ImBz*p*ClPh (**15**)	769.80	>769.80	>769.80	>769.80
4ImBz*p*NO_2_Ph (**16**)	>745.60	>745.60	>745.60	>745.60
4ImAcMe (**17**)	>1031.90	>1031.90	>1031.90	>1031.90
4ImAcPh (**18**)	>821.40	>821.40	>821.40	>821.40
4ImAc*p*ClPh (**19**)	1.40	>737.90	>737.90	>737.90
4ImAc*p*NO_2_Ph (**20**)	<0.68	>715.60	>715.60	>715.60
Nystatin	1.05	>269	>269	>269

## 3. Experimental

### 3.1. General

All common chemicals were purchased from Aldrich and used without further purification. Thiosemicarbazones and hydrazones were prepared according to procedures previously employed by our group [15,19,31,32,33]. Melting points were determined using the digital Mettler Toledo FP90 equipment. Partial elemental analyses were performed on a Perkin Elmer CHN 2400 analyzer. Infrared spectra were recorded on a Perkin Elmer FT-IR Spectrum GX spectrometer using KBr plates (4000–400 cm^−1^). NMR spectra were obtained with a Bruker DPX-200 Avance (200 MHz) spectrometer using DMSO-*d*_6_ as the solvent and TMS as internal reference. X-ray diffraction data collection was performed on an Oxford-Diffraction *GEMINI* diffractometer (LabCri-UFMG) using graphite-Enhance Source Mo Kα radiation (λ = 0.71069 Å or Cu Kα radiation (λ = 1.54180 Å Data integration and scaling of the reflections were performed with the Crysalis suite [34]. Final unit cell parameters were based on the fitting of all reflections positions.

### 3.2. Synthesis of Thiosemicarbazone Derivatives (**1**–**8**)

The imidazole-derived thiosemicarbazones were prepared by reacting equimolar amounts (2 mmol) of 4(5)-imidazole-carboxaldehyde, 4-(1*H*-imidazole-1-yl)-benzaldehyde or 4-(1*H*-imidazole-1-yl)-acetophenone with the suitable thiosemicarbazide using methanol as solvent. The reaction mixture was kept under reflux for 6 h. After cooling to room temperature the resulting solids were filtered off, washed with ethanol and ether and dried *in vacuum*.

*4(5)-Imidazole-carboxaldehyde-thiosemicarbazone (ImT)* (**1**): Yield: 89%. White solid. M.p.: 201–202 °C. Anal. Calc. for C_5_H_7_N_5_S: C, 35.49; H, 4.17; N, 41.39. Found: C, 35.20; H, 4.04; N, 40.83%. FW: 169.21 g mol^−1^. IR (KBr, cm^−1^): ν(NH) 3446s, ν(C=N) 1616s, ν(C=S) 848m. ^1^H-NMR (DMSO-*d*_6_, δ, *E*, *Z* configuration): 13.21 (s, 1H, N4H, *Z*), 12.89 (s, 1H, N1H, *E,Z*), 11.43 (s, 1H, N4H, *E*), 8.28 (s, 1H, N5H_a_, *Z*), 8.21 (s, 1H, N5H_b_, *Z*), 8.08 (s, 1H, H4, *E*), 8.01 (s, 1H, H2, *Z*), 7.91 (s, 1H, N5H_a_, *E*), 7.91 (s, 1H, N5H_b_, *E*), 7.73 (s, 1H, H1, *E*), 7.47 (s, 1H, H1, *Z*), 7.28 (s, 1H, H2, *E*). ^13^C-NMR (DMSO-*d*_6_, δ, *E*, *Z* configuration): 177.80 (C5, *E*), 177.58 (C5, *Z*), 138.18 (C4, *Z*), 135.54 (C3, *E*, *Z*), 135.47 (C4, *E*), 134.76 (C2, *Z*), 131.14 (C2, *E*), 121.56 (C1, *E*), 121.57 (C1, *Z*).

*4(5)-Imidazole-carbaldehyde-N(5)-phenylthiosemicarbazone (ImTPh)* (**2**): Yield: 80%. White solid. Mp.: 219–220 °C. Anal. Calc. for C_11_H_11_N_5_S: C, 53.86; H, 4.52; N, 28.55. Found: C, 53.93; H, 4.52; N, 28.52%. FW: 245.30 g mol^−1^. IR (KBr, cm^−1^): ν(NH) 3358s, ν(C=N) 1635s, ν(C=S) 841m. ^1^H-NMR [DMSO-*d*_6_, δ, *J*(Hz), *E*, *Z* configuration, *thiol*]: 13.41(s, 1H, N4H, *Z*); 12.89 (s, 1H, N1H, *E*, *Z*, *thiol*); 11.78 (s, 1H, N4H, *E*); 10.18 (s, 1H, N5H_b_, *E*); 10.06 (s, 1H, N5H_b_, *Z*); 8.79 (s, 1H, H4, *thiol*); 8.07 (s, 1H, H4, *Z*); 7.89 (s, 1H, H2, *Z*); 7.87 (s, 1H, H1, *thiol*); 7.87 (s, 1H, H2, *thiol*); 7.87 (s, 1H, H4, *E*); 7.86 (t, 2H, H7, H11, 8.56, *E*,*Z*); 7.75 (s, 1H, H1, *E*, *Z*); 7.35 (s, 1H, H2, *E*); 7.61 (t, 2H, H8, H10, 8.55, *E*, *Z*, *thiol*); 7.17 (t, 1H, H9, 7.89, E, *Z*, *thiol*). ^13^C-NMR (DMSO-*d*_6_, δ, *E*, *Z* configuration, *thiol*): 175.47 (C5, *Z*); 175.29 (C5, *E*); 172.05 (C5, *thiol*); 165.20 (C6, *thiol*); 138.99 (C6, *E*, *Z*); 138.84 (C1, *thiol*); 137.57 (C4, *E*); 136.68 (C4, *Z*); 135.74 (C4, *thiol*); 134.70 (C2, *Z*); 135.36 (C3, *E*, *Z*, *thiol*); 131.28 (C2, *E*); 128.70 (C7, C11, *thiol*); 128.61 (C2, *thiol* ); 127.99 (C7, C11, *E*, *Z*); 125.15 (C8, C10, *thiol*); 124.96 (C8, C10, *E*, *Z*); 124.73 (C9, *E*, *Z*); 121.96 (C1, *E*, *Z*); 118.80 (C9, *thiol*). 

*4-(1H-Imidazole-1-yl)-benzaldehyde-thiosemicarbazone (4ImBzT)* (**3**): Yield: 96%. White solid. M.p.: 244–245 °C. Anal. Calc. for C_11_H_11_N_5_S: C, 53.86; H, 4.52; N, 28.55. Found: C, 53.68; H, 4.19; N, 28.75%. FW: 245.30 g mol^−1^. IR (KBr, cm^−1^): ν(NH) 3444s, ν(C=N) 1616s, ν(C=S) 848m. ^1^H-NMR [DMSO-*d*_6_, δ, *J* (Hz)]: 11.53 (s, 1H, N4H); 8.38 (s, 1H, H10); 8.28 (s, 1H, N5H_a_); 8.14 (s, 1H, N5H_b_); 8.02 (s, 1H, H1); 7.98 (d, 2H, H5, H9, 8.30); 7.85 (s, 1H, H3); 7.72 (d, 2H, H6, H8, 8.31); 7.16 (s, 1H, H2). ^13^C-NMR (DMSO-*d*_6_, δ): 177.96 (C11); 141.25 (C1); 137.56 (C4); 135.49 (C10); 132.62 (C7); 129.99 (C2); 128.74 (C6); 128.74 (C8); 120.11 (C5); 120.11 (C9); 117.79 (C3).

*4-(1H-Imidazole-1-yl)-benzaldehyde-N(5)-methylthiosemicarbazone (4ImBzTM)* (**4**): Yield: 76%. White solid. M.p.: 204–206 °C. Anal. Calc. for C_12_H_13_N_5_S: C, 55.58; H, 5.05; N, 27.01. Found: C, 55.88; H, 4.43; N, 26.95%. FW: 259.33 g mol^−1^. IR (KBr, cm^−1^): ν(NH) 3145s, ν(C=N) 1610s, ν(C=S) 835m. ^1^H-NMR [DMSO-*d_6_*, δ, *J* (Hz)]: 11.57 (s, 1H, N4H); 8.60 (s, 1H, N5H_a_); 8.36 (s, 1H, H10); 8.07 (s, 1H, H1); 7.93 (d, 2H, H5, H9, 7.52); 7.82 (s, 1H, H3); 7.72 (d, 2H, H6, H8, 8.53); 7.13 (s, 1H, H2); 3.03 (d, 3H, H12, *4.37*). ^13^C-NMR (DMSO-*d_6_*, δ): 177.67 (C11); 140.49 (C1); 137.49 (C4); 135.49 (C10); 132.69 (C7); 129.99 (C2); 128.58 (C6); 128.58 (C8); 120.10 (C5); 120.10 (C9); 117.78 (C3); 30.81 (C12).

*4-(1H-Imidazole-1-yl)-benzaldehyde-N(5)-phenylthiosemicarbazone (4ImBzTPh)* (**5**): Yield: 83%. White solid. M.p.: 194–196 °C. Anal. Calc. for C_17_H_15_N_5_S: C, 63.53; H, 4.70; N, 21.79. Found: C, 63.03; H, 4.70; N, 21.82%. FW: 321.34 g mol^−1^. IR (KBr, cm^−1^): ν(NH) 3358 s, ν(C=N) 1635s, ν(C=S) 837m. ^1^H-NMR [DMSO-*d*_6_, δ, *J* (Hz)]: 8.38 (s, 1H, H10); 8.20 (s, 1H, H1); 8.06 (d, 2H, H6, H8, 8.41); 7.84 (s, 1H, H3); 7.70 (d, 2H, H5, H9, 8.42); 7.58 (d, 2H, H13, H17, 7.67); 7.57 (t, 1H, H16); 7.38 (t, 2H, H14, H16, 7.57); 7.14 (s, 1H, H2). ^13^C NMR (DMSO-*d*_6_, δ): 176.01 (C11); 141.68 (C1), 139.00 (C12); 137.69 (C4); 135.49 (C10); 132.40 (C7); 130.01 (C2), 129.05 (C8); 129.05 (C6); 127.99 (C14, C16); 125.97 (C13, C17); 125.34 (C15); 120.04 (C5); 120.03 (C9); 117.75 (C3).

*4-(1H-Imidazole-1-yl)-acetophenone-N(5)-phenylthiosemicarbazone (4ImAcT)* (**6**)*:* Yield: 83%. White solid. M.p.: 249–251 °C. Anal. Calc. for C_12_H_13_N_5_S: C, 55.58; H, 5.05; N, 27.01. Found: C, 55.31; H, 4.98; N, 27.43%. FW: 295.33 g mol^−1^. IR (KBr, cm^−1^): ν(NH) 3444s, ν(C=N) 1616s, ν(C=S) 848m. ^1^H-NMR [DMSO-*d*_6_, δ, *J* (Hz)]: 10.27 (s, 1H, N4H); 8.35 (s, 1H, H1); 8.33 (s, 2H, N5H); 8.08 (d, 2H, H6, H8, 8.67); 7.82 (s, 1H, H3); 7.66 (d, 2H, H5, H9, 8.66); 7.12 (s, 1H, H2); 2.32(s, 3H, H12). ^13^CNMR (DMSO-*d*_6_, δ): 178.83 (C11); 145.85 (C4); 137.25 (C7); 135.93 (C10); 135.52 (C1); 130.00 (C2); 128.15 (C6); 128.15 (C8); 119.63 (C5); 119.63 (C9); 117.85 (C3); 13.91 (C12).

*4-(1H-Imidazole-1-yl)-acetophenone-N(5)-methylthiosemicarbazone (4ImAcTM)* (**7**): Yield: 76%. White solid. M.p.: 215–216 °C. Anal. Calc. for C_13_H_15_N_5_S: C, 57.12; H, 5.53; N, 25.62. Found: C, 57.17; H, 5.57; N, 25.55%. FW: 273.36 g mol^−1^. IR (KBr, cm^−1^): ν(NH) 3358s, ν(C=N) 1615s, ν(C=S) 830m. ^1^H-NMR [DMSO-*d*_6_, δ, *J* (Hz)]: 10.28 (s, 1H, N4H); 8.54 (s, 1H, N5H_a_); 8.37 (s, 1H, H1); 8.08 (d, 2H, H6, H8, 8.70); 7.83 (s, 1H, H3); 7.67 (d, 2H, H5, H9, 8.72); 7.13 (s, 1H, H2); 3.05 (d, 3H, H13); 2.32 (s, 3H, H12). ^13^C-NMR (DMSO-*d*_6_, δ): 175.56 (C11); 146.50 (C4); 137.18 (C7); 135.96 (C10); 135.50 (C1); 129.97 (C2); 128.06 (C8); 128.06 (C6); 119.62 (C9); 119.62 (C5); 117.82 (C3); 13.91 (C12); 31.10 (C13).

*4-(1H-Imidazole-1-yl)-acetophenone-N(5)-phenylthiosemicarbazone (4ImAcTPh)* (**8**): Yield: 70%. White solid. M.p.: 191–192 °C. Anal. Calc. for C_18_H_17_N_5_S: C, 64.45; H, 5.11; N, 20.88. Found: C, 64.39, H, 5.98; N, 20.60%. FW: 335.42 g mol^−1^. IR (KBr, cm^−1^): ν(NH) 3286s, ν(C=N) 1612s, ν(C=S) 832m. ^1^H-NMR [DMSO-*d*_6_, δ, *J* (Hz)]: 8.39 (s, 1H, H1); 8.18 (d, 2H, H6, H8, 8.57); 7.85 (s, 1H, H3); 7.70 (d, 2H, H5, H9, 8.60); 7.58 (d, 2H, H14, H18, 7.80); 7.39 (t, 2H, H15, H17, 7.66); 7.23 (t, 1H, H16, 7.38); 7.16 (s, 1H, H2); 2.42 (s, 3H, H12). ^13^C-NMR (DMSO-*d*_6_, δ): 176.99 (C11); 147.89 (C4); 139.13 (C13); 137.35 (C7); 135.78 (C10); 135.52 (C1), 129.89 (C2), 128.40 (C6, C8); 128.06 (C15, C17); 125.97 (C14, C18), 125.39 (C16); 119.64 (C5, C9); 117.84 (C3); 14.33 (C12).

### 3.3. Synthesis of Hydrazone Derivatives (**9**–**20**)

The imidazole-derived hydrazones were prepared by mixing equimolar amounts (2 mmol) of 4(5)-imidazole-carboxaldehyde, 4-(1*H*-imidazole-1-yl)-benzaldehyde and 4-(1*H*-imidazole-1-yl)-acetophenone with the desired hydrazide in methanol with addition of three drops of acetic acid as catalyst. The reaction mixture was kept under reflux for 6 h. After cooling to room temperature the resulting solids were filtered off, washed with ethanol and ether and dried *in vacuum*. In the case of compound **9**, the synthesis was carried out using acetonitrile as solvent.

*4(5)-Imidazole-carboxaldehyde-acetylhydrazone [4(5)ImM]* (**9**): Yield: 76%. White solid. M.p.: 163–164 °C. Anal. Calc. for C_6_H_8_N_4_O: C, 47.63; H, 5.30; N, 36.82. Found C, 47.45; H, 5.52; N, 36.86%. FW: 152.15 g mol^−1^. IR (KBr, cm^−1^): ν(N-H) 3210 s; ν(N-H)_imidazole_ 3097–3004 m; ν(CH_3ass_) 2930 m; ν(CH_3s_) 2864 m; ν(C=O) 1675 s; ν(C=N) 1615 m. ^1^H-NMR (DMSO-*d*_6_, δ, *E*, *Z* configuration, *enol*): 13.30 (s, 1H, N4H, *Z*); 12.93 (s, 1H, N4H, *E*); 12.93 (s, 1H, OH, *enol*); 11.22 (s, 1H, N1H, *enol*); 11.03 (s, 1H, N1H, *E*); 9.76 (s, 1H, N1H, *Z*); 8.10 (s, 1H, H4, *enol*); 8.03 (s, 1H, H4, *Z*), 7.94 (s, 1H, H4, *E*); 7.76 (s, 1H, H1, *Z*); 7.68 (s, 1H, H1, *E*); 7.43 (s, 1H, H1, *enol*); 7.37 (s, 1H, H2, *Z*); 7.24 (s, 1H, H2,*E*, *enol*); 2.02 (s, 3H, H6, *Z*), 2.16 (s, 3H, H6, *E*), 1.94 (s, 3H, H6, *enol*). ^13^C-NMR (DMSO-*d*_6_, *δ*, *E*, *Z* configuration, *enol*): 171.89 (C5, *E*); 171.57 (C5, *Z*); 168.35 (C5, *enol*); 139.55 (C4, Enol); 137,0 (C4, *E*); 136.33 (C4, *Z*); 137.80 (C2, *enol*); 134.13 (C2, *Z*); 131.80 (C2, *E*); 137.36 (C3, *Z*); 121.25 (C3, *enol*); 120.19 (C3, *E*); 135.74 (C1, *E*); 135.53 (C1, *enol*); 22.15 (C6, *Z*); 21.54 (C6, *enol*); 20.08 (C6, *E*).

*4(5)-Imidazole-carboxaldehyde-benzoylhydrazone [4(5)ImPh]* (**10**): Yield: 81%. White solid. M.p.: 276–278 °C. Anal. Calc. for C_11_H_10_N_4_O: C, 61.67; H, 4.71; N, 26.15. Found: C, 61.80; H, 4.68; N, 26.06%. FW: 214.22 g mol^−1^. IR (KBr, cm^−1^): ν(N-H) 3213 s; ν(N-H)_imidazole_ 3100–3027 m; ν(C=O) 1644 s; ν(C=N) 1626 m. ^1^H-NMR [DMSO-*d*_6_, δ, *J*(Hz)]: 14.29 (s, 1H, N4H); 8.37 (s, 1H, N1H); 8.11 (s, 1H, H4); 7.89 (d, 2H, H7, H11, *6.86*); 7.75 (s, 1H, H1); 7.58 (m, 1H, H9); 7.53 (m, 2H, H8, H10, *6.91*); 7.51(s, 1H, H2). ^13^C-NMR (DMSO-*d*_6_, δ): 162.81 (C5); 136.61 (C4); 135.67 (C2); 135.03 (C1); 133.55 (C6); 131.54 (C9); 128.14 (C8, C10); 127.44 (C7, C11); 121.26 (C3).

*4(5)-Imidazole-carboxaldehyde-para-chloro-benzoylhydrazone [4(5)ImpClPh]* (**11**): Yield: 56%. White solid. M.p.: 277–280 °C. Anal. Calc. for C_11_H_9_ClN_4_O: C, 53.13; H, 3.65; N, 22.53. Found C, 53.08; H, 3.59; N, 22.32%. FW: 248.67 g mol^−1^. IR (KBr, cm^−1^): ν(N-H) 3200 s; ν(N-H)_imidazole_ 3100–3058 m; ν(C=O) 1660 s; ν(C=N) 1615 m. ^1^H-NMR [DMSO-*d*_6_, δ, *J*(Hz)]: 14.34 (s, 1H, N4H); 8.39 (s, 1H, N1H); 8.12 (s, 1H, H4); 7.95 (d, 2H, H7, H11, *8.55*); 7.79 (s, 1H, H1); 7.65(d, 2H, H8, H10, *8.64*); 7.55(s, 1H, H2). ^13^C-NMR (DMSO-*d*_6_, δ): 161.26 (C5); 136.50 (C4); 136.61 (C9); 135.99 (C2); 135.59 (C1); 132.23 (C6); 128.99 (C8, C10); 128.81 (C7, C11); 121.61 (C3).

*4(5)-Imidazole-carboxaldehyde-para-nitro-benzoylhydrazone [4(5)ImpNO_2_Ph]* (**12**): Yield: 76%. Yellow solid. M.p.: >300 °C. Anal. Calc. for C_11_H_9_N_5_O_3_: C, 50.97; H, 3.50; N, 27.02. Found: C, 50.58; H, 3.43; N, 26.96%; mol wt, 259.22 g mol^−1^. IR (KBr, cm^−1^): ν(N-H) 3159 s; ν(N-H)_imidazole_ 3127–3063 m; ν(C=O) 1687 s; ν(C=N) 1619 m. ^1^H-NMR [DMSO-*d*_6_, δ, *J*(Hz)]: 14.54 (s, 1H, N4H); 8.41 (d, 2H, H8, H10, *6.89*); 8.37 (s, 1H, N1H); 8.18 (m, 2H, H7, H11); 8.13 (m, 1H, H4); 7.82 (s, 1H, H1); 7.61(s, 1H, H2). Due the low solubility of compound **12** was impossible to record the ^13^C-NMR spectrum.

*4-(1H-Imidazole-1-yl)-benzaldehyde-acetylhydrazone (4ImBzM)* (**13**): Yield: 65%. White solid. M.p.: 148–150 °C. Anal. Calc. for C_12_H_12_N_4_O: C, 63.14; H, 5.30; N, 24.55. Found C, 63.07; H, 5.23; N, 24.66%. FW: 228.25 g mol^−1^. IR (KBr, cm^−1^): ν(N-H) 3171 s; ν(CH_3ass_) 2978m; ν(CH_3s_) 2863 m; ν(C=O) 1659 s; ν(C=N) 1618 m. ^1^H NMR [DMSO-*d*_6_, δ, *J* (Hz), *keto*, *enol* tautomers]: 11.49 (s, 1H, N4H, *keto*); 11.35 (s, 1H, OH, *enol*); 8.36 (s, 1H, H1, *keto*); 8.20 (s, 1H, H10, *keto*); 8.03 (s, 1H, H10, *enol*); 7.83 (s, 1H, H3, *keto*); 7.81 (m, 2H, H6, H8, *keto*); 7.76 (m, 2H, H5, H9, *keto*); 7.15 (s, 1H, H2, *keto*); 2.23 (s, 3H, H12, *enol*); 1.99 (s, 3H, H12, *keto*). ^13^C-NMR (DMSO-*d*_6_, δ, *keto*, *enol* tautomers): 172.00 (C11, *keto*); 165.67 (C11, *enol*); 144.49 (C10, *keto*); 141.38 (C10, *enol*); 137.63 (C4, *keto*); 135.45 (C1, *keto*); 132.70 (C7, *keto*); 130.02 (C2, *keto*); 127.97 (C6, C8, *keto*); 120.30 (C5, C9, *keto*); 117.75 (C3, *keto*); 21.58 (C12, *keto*), 20.12 (C12, *enol*).

*4-(1H-Imidazole-1-yl)-benzaldehyde-benzoylhydrazone (4ImBzPh)* (**14**): Yield: 84%. White solid. M.p.: 250-252 °C. Anal. Calc. for C_17_H_14_N_4_O: C, 70.33; H, 4.86; N, 19.30. Found: C, 70.10; H, 4.70; N, 19.47%. FW: 290.32 g mol^−1^. IR (KBr, cm^−1^): ν(N-H) 3195 s; ν(C=O) 1678 s; ν(C=N) 1610 m. ^1^H-NMR [DMSO-*d*_6_, δ, *J*(Hz)]: 11.95 (s, 1H, N4H); 8.52 (s, 1H, H10); 8.38 (s,1H, H1); 7.96 (d, 2H, H13, H17, 7.55); 7.90 (m, 2H, H6, H8); 7.86 (s, 1H, H3); 7.80 (m, 2H, H5, H9, 8.46); 7.60 (t, 3H, H14, H15, H16, 7.58); 7.16 (s, 1H, H2). ^13^C-NMR (DMSO-*d*_6_, δ): 163.12 (C11); 146.62 (C10); 137.77 (C4); 135.45 (C1); 133.30 (C12); 132.66 (C7); 131.70 (C15); 130.05 (C2); 128.41 (C6, C8 and C14, C16); 127.58 (C13, C17); 120.27 (C5, C9); 117.72 (C3).

*4-(1H-Imidazole-1-yl)-benzaldehyde-para-chloro-benzoylhydrazone (4ImBzpClPh)* (**15**): Yield: 92%. White solid. M.p.: 244–245 °C. Anal. Calc. for C_17_H_13_ClN_4_O: C, 62.87; H, 4.03; N, 17.25. Found: C, 62.94; H, 4,01; N, 17.05%. FW: 324.76 g mol^−1^. IR (KBr, cm^−1^): ν(N-H) 3189 s; ν(C=O) 1678 s; ν(C=N) 1608 m. ^1^H-NMR [DMSO-*d*_6_, δ, *J* (Hz)]: 12.01 (s, 1H, N4H); 8.51 (s, 1H, H10); 8.38 (s,1H, H1); 7.97 (d, 2H, H13, H17, 8.27); 7.89 (d, 2H, H6, H8, 8.50); 7.85 (m, 1H, H3); 7.78 (m, 2H, H14, H16, 8.49); 7.64 (d, 2H, H5, H9, 8.29); 7.16 (s, 1H, H2). ^13^C-NMR (DMSO-*d*_6_, δ): 162.03 (C11); 146.96 (C10); 137.84 (C15); 136.55 (C4); 135.45 (C1); 132.54 (C7); 131.97 (C12); 130.05 (C2); 129.49 (C6, C8); 128.49 (C13, C17 and C14, C16); 120.29 (C5, C9); 117.71 (C3).

*4-(1H-Imidazole-1-yl)-benzaldehyde-para-nitro-benzoylhydrazine (4ImBzpNO_2_Ph)* (**16**): Yield: 96%. Yellow solid. M.p.: 261–263 °C. Anal. Calc. for C_17_H_13_N_5_O_3_: C, 60.89; H, 3.91; N, 20.89. Found: C, 60.51; H, 3.92; N, 20.78%. FW: 335.31 g mol^−1^. IR (KBr, cm^−1^): ν(N-H) 3194 s; ν(C=O) 1687 s; ν(C=N) 1606 m. ^1^H NMR [DMSO-*d*_6_, δ, *J* (Hz)]: 12.22 (s, 1H, N4H); 8.52 (s, 1H, H10); 8.41 (d, 2H, H14, H16, 7.12); 8.38 (s,1H, H1); 8.18 (d, 2H, H13, H17, 8.49); 7.92 (d, 2H, H6, H8, 8.58); 7.85 (s, 1H, H3); 7.81 (d, 2H, H5, H9, 8.22); 7.16 (s, 1H, H2). ^13^C NMR (DMSO-*d*_6_, δ): 161.46 (C11); 147.72 (C10); 149.19 (C15); 138.93 (C4); 137.99 (C12); 135.42 (C1); 132.34 (C7); 130.11 (C2); 129.10 (C13, C17); 128.65 (C6, C8); 123.27 (C14, C16); 120.30 (C5, C9); 117.70 (C3).

*4-(Imidazole-1-yl)-acetophenone-acetylhydrazone (4ImAcM)* (**17**): Yield: 68%. White solid. M.p.: 210–211 °C. Anal. Calc. for C_13_H_14_N_4_O: C 64.45; H, 5.82; N, 23.13. Found: C, 64.30; H, 5.75; N, 23.24%. FW: 242.27 g mol^−1^. IR (KBr, cm^−1^): ν(N-H) 3183 s; ν(CH_3ass_) 2933m; ν(CH_3s_) 2873 m; ν(C=O) 1674 s; ν(C=N) 1615 m. ^1^H-NMR [DMSO-*d*_6_, δ, *J* (Hz), *keto*, *enol* tautomers]: 10.51 (s, 1H, N4H, *keto*); 10.41 (s, 1H, OH, *enol*); 8.33 (s, 1H, H1, *keto*); 7.90 (d, 2H, H6, H8, *8.64*, *keto*); 7.80 (s, 1H, H3, *keto*); 7.70 (d, 2H, H5, H9, *8.64*, *keto*); 7.14 (s, 1H, H2, *keto*); 2.29 (s, 3H, H12, *enol*); 2.27 (s, 3H, H12, *keto*); 2.26 (s, 3H, H13, *enol*); 2.06 (s, 3H, H13, *keto*). ^13^C-NMR (DMSO-*d*_6_, δ, *keto*, *enol* tautomers): 171.82 (C11, *keto*); 166.13 (C11, *keto*); 149.31 (C10, *enol*); 145.78 (C10, *keto*); 137.14 (C7, *enol*); 137.00 (C7, *keto*); 136.53 (C4, *keto*); 135.42 (C1, *keto*); 129.96 (C2, *keto*); 127.60 (C6, C8, *enol*); 127.28 (C6, C8, *keto*), 119.84 (C5, C9, *keto*); 119.70 (C5, C9, *enol*); 117.76 (C3, *keto*); 20.84 (C13, *keto*); 21.65 (C13, *enol*); 13.38 (C12, *keto*); 13.83 (C12, *enol*).

*4-(Imidazole-1-yl)-acetophenone-benzoylhydrazone (4ImAcPh)* (**18**): Yield: 92%. White solid. M.p.: 242–243 °C. Anal. Calc. for C_18_H_15_N_4_O: C, 71.04; H, 5.30; N, 18.41. Found: C, 71.10; H, 5.47; N, 18.32%. FW: 304.34 g mol^−1^. IR (KBr, cm^−1^): ν(N-H) 3139 s; ν(C=O) 1663 s; ν(C=N) 1614 m. ^1^H-NMR [DMSO-*d*_6_, δ, *J* (Hz)]:10.84 (s, 1H, N4H); 8.36 (s, 1H, H1); 7.95 (d, 2H, H6, H8, 6.36); 7.89 (d, 2H, H14, H18, 6.62); 7.83 (s, 1H, H3); 7.74 (d, 2H, H5, H9, 8.07); 7.56 (t, 3H, H15, H16, H17, 7.98); 7.14 (s, 1H, H2); 2.40 (s, 3H, H12).^13^C-NMR (DMSO-*d*_6_, δ): 168.03 (C11); 153.79 (C10); 137.37 (C7); 136.29 (C4); 135.43 (C1); 134.02 (C13); 131.23 (C16); 129.88 (C2); 128.05 (C15, C17); 127.66 (C6, C8); 127.62 (C14, C18); 117.72 (C3); 119.75 (C5, C9); 14.49 (C12).

*4-(Imidazole-1-yl)acetophenone-para-chloro-benzoylhydrazone (4ImAcpClPh)* (**19**): Yield: 96%. White solid. M.p.: 240–243 °C. Anal. Calc. for (C_18_H_15_ClN_4_O): C, 63.81; H, 4.46; N, 16.54. Found: C, 63.79; H, 4.38; N, 16.99%. FW: 338.79 g mol^−1^. IR (KBr, cm^−1^): ν(N-H) 3240 s; ν(C=O) 1661 s; ν(C=N) 1611 m. ^1^H-NMR [DMSO-*d*_6_, δ, *J* (Hz)]:10.91 (s, 1H, N4H); 8.36 (s, 1H, H1); 7.92 (m, 4H, H6, H8, H14, H18); 7.83 (s, 1H, H3); 7.75 (d, 2H, H5, H9, 7.60); 7.60 (d, 2H, H15, H17, 8.17); 7.14 (s, 1H, H2); 2.40 (s, 3H, H12).^13^C-NMR (DMSO-*d*_6_, δ): 167.27 (C11); 155.34 (C10); 141.40 (C16); 137.49 (C7); 136.19 (C4); 135.46 (C1); 132.66 (C13); 130.10 (C2); 130.01 (C6, C8); 128.27 (C15, C17); 127.86 (C14, C18); 119.76 (C5, C9); 117.76 (C3); 14.50 (C12).

*4-(Imidazole-1-yl)-acetophenone-para-nitro-benzoylhydrazone (4ImAcpNO_2_Ph)* (**20**): Yield: 76%. Yellow solid. M.p.: 279–281 °C. Anal. Calc. for C_18_H_15_N_5_O_3_: C, 61.89; H, 4.33; N, 20.05. Found: C, 61.72; H, 4.28; N, 20.10%. FW: 349.34 g mol^−1^. IR (KBr, cm^−1^): ν(N-H) 3136 s; ν(C=O) 1665 s; ν(C=N) 1616 m. Due to the low solubility of the compound it was impossible to record its ^1^H and ^13^C-NMR spectrum.

### 3.4. X-ray Crystallography

Crystals of ImT∙H_2_O (**1a)**, ImTPh (**2**), 4ImBzT (**3**), 4ImBzTM (**4**), 4ImBzTPh (**5**), 4(5)Im*p*ClPh∙H_2_O (**11a**), 4ImBzPh (**14**) and 4ImBz*p*ClPh (**15**) were mounted on Mitgen loops in random orientations and used for data collection. The structures were solved by direct methods using SHELXS-97 [35] and refined by full-matrix least-squares techniques against *F*^2^ using SHELXL-97 [36]. Positional and anisotropic atomic displacement parameters were refined for non-hydrogen atoms. Although some hydrogen atoms could be identified in a Fourier difference map, in the final model they were geometrically positioned and refined using a riding model. Molecular graphics were obtained from ORTEP [37,38]. Crystals of **11a** have shown to be twinned. Moreover, the crystal structure presented disordered water molecule in four different positions with equal site occupation factor of 0.25; the corresponding oxygen atoms were refined isotropically.

CCDC 903502, CCDC 952430, CCDC 903501, CCDC 952431, CCDC 952432, CCDC 903499, CCDC 952433 and CCDC 903500 contain the supplementary crystallographic data for **1a**, **2**–**5**, **11a**, **14** and **15**, respectively. These data can be obtained free of charge via http://www.ccdc.cam.ac.uk/data_request/cif, or from the Cambridge Crystallographic Data Centre, 12 Union Road, Cambridge CB2 1EZ, UK; fax: +44-1223-336-033; or e-mail:deposit@ccdc.cam.ac.uk.

### 3.5. Biological Assays

Filamentous fungi, *Cladosporium cladosporioides* (LABB 6) and *Aspergillus flavus* (LABB44) were from Biotechnology and Bioassays Laboratory (LABB, Chemistry Department, Universidade Federal de Minas Gerais, Belo Horizonte, Brazil) and were maintained in potato dextrose agar (PDA) under refrigeration at 7 °C. *Candida glabrata* (ATCC 2001) and *Candida albicans* (ATCC 18804) were maintained in broth heart infusion (BHI). For the experiments, fungi were grown at room temperature in PDA or BHI until abundant sporulation. Spores were harvested and suspended in sterile water, counted on a Neubauer chamber and diluted to give a suspension containing a final concentration of 5 × 10^3^ spores/mL. An initial screening was carried out in microtiter plates, for all compounds, in duplicate, using a concentration of 100 µg/well (in DMSO). The final concentrations of DMSO in the assays did not exceed 2%. Positive (inoculum plus medium) and negative (inoculum plus reference compound nystatin) controls were run simultaneously. Extracts showing activity in the screening step were also tested in the microdilution assay in 12 serial concentrations (250, 125, 62.5, 31.3, 15.6, 7.81, 3.91, 1.95, 0.98, 0.49, 0.24 and 0.12 µg/mL), in duplicate. Fungal inhibition was assessed by using a microplate TP-reader (Thermoplate, Palm City, FL, USA). The minimum inhibitory concentration (MIC) value was defined as the lowest concentration of the compound showing 100% of fungal growth inhibition after incubation time (48 h). MIC_50_ values were assessed and express the lowest concentration of the compounds able to inhibit 50% of fungal growth. Experiments were carried out according to Zacchino and Gupta [39].

## 4. Conclusions

The imidazole-derived thiosemicarbazones were inactive against all fungi strains except for compounds **1** and **3** which proved to be selectively active against *C*. *cladosporioides*. In general the imidazole-derived hydrazones showed antifungal activity against *C. glabrata and C*. *cladosporioides.* Compounds **19** and **20** were selectively active against *C*. *glabrata.* All active compounds were more effective than the control drug nystatin. As already mentioned, selectivity is a very important property of an antifungal, which allows an optimum exploitation of its capacity without affecting beneficiating microorganisms. Hence, some of the studied compounds could constitute novel antifungal drug candidate prototypes.

## Supplementary Materials

Supplementary materials can be accessed at: http://www.mdpi.com/1420-3049/18/10/12645/s1.
