# Supplementary material for: Structural Studies and Investigation on the Activity of Imidazole-Derived Thiosemicarbazones and Hydrazones against Crop-Related Fungi
Source: Molecules. 2013 Oct 14;18(10):12645–62. doi: 10.3390/molecules181012645 (PMC6270485; doi:10.3390/molecules181012645)
Supplement: Supplementary file 1 [file molecules-18-12645-s001.docx]

**Supplementary Materials**

**Table S1.** Crystal data and reﬁnement results for the imidazole-derived thiosemicarbazones ImT∙H_2_O (**1a)**, ImTPh (**2**), 4ImBzT (**3**), 4ImBzTM (**4**) and 4ImBzTPh (**5**).

| **Compound** | | **1a** | **2** | **3** | **4** | **5** |
| --- | --- | --- | --- | --- | --- | --- |
| Empirical formula | | C_5_H_9_N_5_OS | C_11_H_11_N_5_S | C_11_H_11_N_5_S | C_12_H_13_N_5_S | C_17_H_15_N_5_S |
| Formula weight (g mol^-1^) | | 187.23 | 245.31 | 245.31 | 259.33 | 321.40 |
| Crystal system | | Monoclinic | Monoclinic | Monoclinic | Triclinic | Monoclinic |
| Crystal size (mm) | | 0.40 × 0.34 × 0.20 | 0.34 × 0.28 × 0.12 | 0.38 × 0.32 × 0.20 | 0.36 × 0.32 × 0.19 | 0.50 × 0.12 × 0.08 |
| Temperature (K) | | 293(2) | 293(2) | 293(2) | 293(2) | 293(2) |
| Wavelength (Å) | | 0.71073 | 0.71073 | 0.71073 | 0.71073 | 0.71073 |
| Space group | | *P* 2_1_/c | *P* 2_1_/c | *P* 2_1_/c | *P* -1 | *P* 2_1_/c |
| Lattice  parameters | a (Å) | 10.9469(5) | 11.2820(3) | 9.5673(4) | 8.94483(4) | 14.1315(5) |
|  | b (Å) | 11.2289(4) | 7.5559(2) | 15.2445(4) | 9.0775(3) | 4.6698(2) |
|  | c (Å) | 7.1883(3) | 13.8018(3) | 8.6535(3) | 16.6341(5) | 23.9591(7) |
|  | α (°) | 90 | 90 | 90 | 91.871(3) | 90 |
|  | β (°) | 104.813(4) | 97.270(2) | 112.726(4) | 92.004(3) | 94.220(3) |
|  | γ (°) | 90 | 90 | 90 | 107.139(3) | 90 |
| V (Å^3^) | | 854.23(6) | 1167.09(5) | 1164.12(7) | 1289.06(8) | 1576.80(10) |
| Z | | 4 | 4 | 4 | 4 | 4 |
| Density calculated (mg m^-3^) | | 1.456 | 1.396 | 1.400 | 1.336 | 1.354 |
| Absorption coeﬃcient (mm^-1^) | | 0.340 | 0.262 | 0.262 | 0.241 | 0.212 |
| *F*(000) | | 392 | 512 | 512 | 544 | 672 |
| θ range for data collection (°) | | 3.45 to 26.37 | 2.98 to 26.37 | 2.88 to 26.37 | 2.81 to 26.37 | 4.31 to 26.37 |
| Limiting indices | | −8 ≤ h ≤ 13  −14 ≤ k ≤ 11  −8 ≤ l ≤8 | −11 ≤ h ≤ 14  −9 ≤ k ≤ 9  −17 ≤ l ≤ 17 | −11 ≤ h ≤ 11  −19 ≤ k ≤ 18  −10 ≤ l ≤ 10 | −9 ≤ h ≤ 11  −11 ≤ k ≤ 11  −20 ≤ l ≤ 15 | −17 ≤ h ≤ 17 −5 ≤ k ≤ 5  −29 ≤ l ≤ 27 |
| Reﬂections collected/unique [Rint] | | 3944/1737 [0.0188] | 5399/2390 [0.0186] | 10109/2375 [0.0282] | 11036/5257 [0.0206] | 8929/3208 [0.0567] |
| Final R indices  [I >2 σ(I)] | | R1 = 0.0336  wR2 = 0.0834 | R1 = 0.0341  wR2 = 0.0802 | R1 = 0.0333  wR2 = 0.0939 | R1 = 0.0439  wR2 = 0.1230 | R1 = 0.0502  wR2 = 0.1316 |
| R indices (all data) | | R1 = 0.0484  wR2 = 0.0876 | R1 = 0.0555  wR2 = 0.0843 | R1 = 0.0429  wR2 = 0.0974 | R1 = 0 .0708  wR2 = 0.1325 | R1 = 0 .0755  wR2 = 0.1405 |
| Completeness to θ = 26.3^o^ | | 99.9% | 100% | 99.9% | 99.8% | 99.7% |
| Absorption correction | | multiscan | multiscan | multiscan | multiscan | multiscan |
| Data/restraints/parameters | | 1737/3/117 | 2390/0/154 | 2375/0/155 | 5257/0/326 | 3208/0/208 |
| Goodness-of-fit on *F^2^* | | 0.980 | 0.978 | 1.087 | 1.056 | 0.925 |
| Largest diﬀerence in peak and hole (e Å^-3^) | | 0.251 and  −0.232 | 0.173 and −0.208 | 0.190 and  −0.270 | 0.251 and −0.252 | 0.391 and −0.240 |

**Table S2.** Selected bond lengths (Å) and angles (°) for ImT∙H_2_O (**1a**), ImTPh (**2**), 4ImBzT (**3**), 4ImBzTM (**4**) and 4ImBzTPh (**5**).

| Bonds | **1a** | **2** | Bonds | **3** | **4** | | **5** |
| --- | --- | --- | --- | --- | --- | --- | --- |
| C1–N1 | 1.344(2) | 1.349(2) | C1–N1 | 1.3083(19) | 1.312(2) | 1.308(2) | 1.298(4) |
| C3–C1 | 1.358(2) | 1.360(2) | C3–C2 | 1.356(2) | 1.350(3) | 1.345(3) | 1.337(4) |
| N1–C2 | 1.330(2) | 1.336(2) | N1–C2 | 1.375(2) | 1.360(3) | 1.363(3) | 1.325(4) |
| C2–N2 | 1.316(2) | 1.306(2) | C3–N2 | 1.3726(19) | 1.368(3) | 1.372(3) | 1.332(4) |
| N2–C3 | 1.386(2) | 1.3836(18) | N2–C1 | 1.3541(19) | 1.359(2) | 1.365(2) | 1.340(4) |
| C3–C4 | 1.439(2) | 1.443(2) | C7–C10 | 1.4632(19) | 1.463(3) | 1.468(3) | 1.461(3) |
| C4–N3 | 1.277(2) | 1.2826(19) | C10–N3 | 1.2739(19) | 1.274(2) | 1.279(3) | 1.270(2) |
| N3–N4 | 1.3755(17) | 1.3691(15) | N3–N4 | 1.3760(16) | 1.382(2) | 1.380(2) | 1.366(2) |
| C5–N4 | 1.3447(19) | 1.3560(19) | C11–N4 | 1.3476(18) | 1.352(2) | 1.358(2) | 1.349(2) |
| C5–N5 | 1.317(2) | 1.3409(18) | C11–N5 | 1.3146(19) | 1.316(2) | 1.319(3) | 1.339(2) |
| C5–S1 | 1.6884(16) | 1.6680(15) | C11–S1 | 1.6911(14) | 1.6931(18) | 1.6856(18) | 1.673(2) |
| Angles | **1a** | **2** | Angles | **3** | **4** | | **5** |
| C3–C4–N3 | 120.72(16) | 129.85(14) | C7–C10–N3 | 122.09(14) | 120.69(19) | 119.53(19) | 120.07(19) |
| C4–N3–N4 | 116.00(14) | 117.03(12) | N4–N3–C10 | 115.41(13) | 116.00(17) | 116.52(18) | 116.90(17) |
| C5–N4–N3 | 119.38(13) | 120.26(12) | N3–N4–C11 | 120.11(12) | 119.87(17) | 118.83(17) | 120.30(16) |
| N4–C5–S1 | 119.25(12) | 118.97(11) | N4–C11–S1 | 119.26(11) | 118.20(15) | 118.58(15) | 120.45(15) |
| N5–C5–N4 | 117.31(14) | 113.76(13) | N5–C11–N4 | 117.84(12) | 117.48(16) | 116.75(17) | 115.75(17) |
| N5–C5–S1 | 123.43(12) | 127.25(12) | N5–C11–S1 | 122.90(11) | 124.31(15) | 124.67(16) | 123.79(15) |

**Table S3.** Crystal data and reﬁnement results for the imidazole-derived hydrazones 4(5)Im*p*ClPh∙H_2_O (**11a**), 4ImBzPh (**14**) and 4ImBz*p*ClPh (**15**).

| Compound | | **11a** | **14** | **15** |
| --- | --- | --- | --- | --- |
| Empirical formula | | C_11_H_9_ClN_4_O_2_ | C_17_H_14_N_4_O | C_17_H_13_ClN_4_O |
| Formula weight (g mol^-1^) | | 264.67 | 290.32 | 324.76 |
| Crystal system | | Monoclinic | Monoclinic | Monoclinic |
| Crystal size (mm) | | 0.26 × 0.20 × 0.08 | 0.30 × 0.26 × 0.10 | 0.37 × 0.32 × 0.30 |
| Temperature (K) | | 293(2) | 293(2) | 293(2) |
| Wavelength (Å) | | 1.54184 | 1.54184 | 0.71073 |
| Space group | | *P* 2_1_/n | *P* 2_1_/c | *P* 2_1_/c |
| Lattice  parameters | a (Å) | 10.9469(5) | 8.2540(9) | 9.5673(4) |
|  | b (Å) | 11.2289(4) | 19.9337(17) | 15.2445(4) |
|  | c (Å) | 7.1883(3) | 9.1188(14) | 8.6535(3) |
|  | α (°) | 90 | 90 | 90 |
|  | β (°) | 104.813(4) | 106.113(14) | 112.726(4) |
|  | γ (°) | 90 | 90 | 90 |
| V (Å^3^) | | 1191.9(16) | 1441.4(3) | 1490.1(3) |
| Z | | 4 | 4 | 4 |
| D. calc (mg/m^3^) | | 1.475 | 1.338 | 1.448 |
| Absorption coeﬃcient (mm^-1^) | | 2.864 | 0.703 | 0.266 |
| F(000) | | 544 | 608 | 672 |
| θ range for data collection (°) | | 3.64 to 62.73 | 4.44 to 62.75 | 2.80 to 26.37 |
| Limiting indices | | −2 ≤ h ≤ 4  −16 ≤ k ≤ 16  −24 ≤ l ≤ 24 | −9 ≤ h ≤ 4 −20 ≤ k ≤ 22 −8 ≤ l ≤ 10 | −8 ≤ h ≤ 10  −22 ≤ k ≤ 25  v11 ≤ l ≤ 11 |
| Reﬂections collected/unique [Rint] | | 2379/ 1907  [0.0000] | 4085/ 2246  [0.0184] | 8389/3044  [0.0218] |
| Final R indices  [I > 2 σ(I)] | | R1 = 0.0665  wR2 = 0.1845 | R1 = 0.0528  wR2 = 0.1284 | R1 = 0.0347  wR2 = 0.0916 |
| R indices (all data) | | R1 = 0.0869  wR2 = 0.2031 | R1 = 0.0804  wR2 = 0.1448 | R1 = 0.0500  wR2 = 0.0960 |
| Completeness to θ full | | 96.3% (θ = 62.73) | 97.0% (θ = 62.75) | 99.9% (θ = 26.37) |
| Absorption correction | | Multi-scan | Multi-scan | Multi-scan |
| Data/restraints/parameters | | 1907/0/171 | 2246/0/200 | 3044/0/208 |
| Goodness-of-fit on *F^2^* | | 1.056 | 1.054 | 1.024 |
| Largest diﬀerence in peak and hole (e Å^-3^) | | 0.288 and 0.224 | 0.314 and −0.159 | 0.184 and −0.219 |

**Table S4.** Selected bond lengths (Å) and angles (°) for 4(5)Im*p*ClPh∙H_2_O (**11a**), 4ImBzPh (**14**) and 4ImBz*p*ClPh (**15**).

| Bonds | **11a** | Bonds | **14** | **15** |
| --- | --- | --- | --- | --- |
| C1–N1 | 1.349(6) | C1–N1 | 1.308(3) | 1.3084(18) |
| C3–C1 | 1.372(6) | C3–C2 | 1.338(4) | 1.344(2) |
| N1–C2 | 1.337(6) | N1–C2 | 1.375(4) | 1.3698(19) |
| C2–N2 | 1.309(6) | C3–N2 | 1.363(3) | 1.3709(18) |
| N2–C3 | 1.388(5) | N2–C1 | 1.363(3) | 1.3585(18) |
| C3–C4 | 1.434(6) | C7–C10 | 1.451(4) | 1.4635(19) |
| C4–N3 | 1.291(5) | C10–N3 | 1.288(3) | 1.2717(18) |
| N3–N4 | 1.374(4) | N3–N4 | 1.358(3) | 1.3695(15) |
| C5–N4 | 1.355(5) | C11–N4 | 1.368(3) | 1.3577(17) |
| C5–C6 | 1.494(5) | C11–C12 | 1.479(4) | 1.4931(18) |
| C5–O1 | 1.218(5) | C11–O1 | 1.225(3) | 1.2215(16) |
| Angles | **11a** | Angles |  | **15** |
| C3–C4–N3 | 130.8(4) | C7–C10–N3 | 120.7(2) | 120.00(12) |
| C4–N3–N4 | 114.5(4) | C10–N3–N4 | 115.9(2) | 117.23(11) |
| C5–N4–N3 | 119.1(3) | C11–N4–N3 | 119.5(2) | 117.78(11) |
| C6–C5–N4 | 115.2(4) | C12–C11–N4 | 115.3(2) | 116.14(12) |
| C6–C5–O1 | 121.8(4) | C12–C11–O1 | 123.1(3) | 121.25(13) |
| N4–C5–O1 | 123.0(4) | N4–C11–O1 | 121.6(3) | 122.59(13) |
